# RNF7 promotes glioma growth via the PI3K/AKT signalling axis

**DOI:** 10.1111/jcmm.17656

**Published:** 2022-12-28

**Authors:** Nan Tang, Kai Zhu, Cheng Jiang, Zhiyong Xiong, Qiangping Wang, Junjun Li, Weiming Xu

**Affiliations:** ^1^ Department of Neurosurgery, Union Hospital, Tongji Medical College Huazhong University of Science and Technology Wuhan China

**Keywords:** apoptosis, cell cycle, glioma, proliferation, RNF7/PI3K/AKT

## Abstract

RNF7 has been reported to play critical roles in various cancers. However, the underlying mechanisms of RNF7 in glioma development remain largely unknown. Herein, the expression level of RNF7 was examined in tissues by quantitative real‐time PCR, Western blotting and immunohistochemistry. The effect of RNF7 on glioma progression was measured by performing CCK‐8 and apoptosis assays, cell cycle‐related experiments and animal experiments. The effect of RNF7 on PI3K/AKT signalling pathway was tested by Western blotting. First, we found that RNF7 was upregulated in tumour tissue compared with normal brain tissue, especially in high‐grade glioma, and the high expression of RNF7 was significantly related to tumour size, Karnofsky Performance Scale score and a poor prognosis. Second, RNF7 overexpression facilitated tumour cell cycle progression and cell proliferation and suppressed apoptosis. Conversely, RNF7 knockdown suppressed tumour cell cycle progression and cell proliferation and facilitated apoptosis. Furthermore, follow‐up mechanistic studies indicated that RNF7 could facilitate glioma cell proliferation and cell cycle progression and inhibit apoptosis by activating the PI3K/AKT signalling pathway. This study shows that RNF7 can clearly promote glioma cell proliferation by facilitating cell cycle progression and inhibiting apoptosis by activating the PI3K/AKT signalling pathway. Targeting the RNF7/PI3K/AKT axis may provide a new perspective on the prevention or treatment of glioma.

## 1 INTRODUCTION

Malignant glioma, the most common intracranial tumour, is highly aggressive, heterogeneous, invasive and neurologically destructive.^1^, ^2^ Despite continued advances in multimodal therapy, such as surgery, combined chemoradiotherapy, TTF and molecular targeted therapy, the clinical outcomes remain poor, and the 5‐year survival rate has not noticeably improved.^3^ Due to the rapid growth and highly invasive nature of gliomas, infiltration into surrounding normal brain tissue is favoured and gliomas easily recur after therapy.^4^ Hence, it seems urgent and imperative to uncover the molecular mechanisms that regulate tumour growth and explore novel targets for early diagnosis and treatment.

High‐throughput proteomics was used to screen candidates worthy of further pursuit. The high‐throughput screen indicated that RNF7 might regulate glioma development. RNF7 is a highly conserved ring finger protein and critical subunit of SKP1‐cullin/CDC53‐F box protein ubiquitin ligases, which regulate cell cycle progression and signal transduction. An increasing number of studies on RNF7 biological functions have been reported recently. Gianluca Telesio et al. reported that RNF7 interacts with CARMA2 and further activates CARMA2 signalling by influencing the ubiquitination state of MALT1 and the NF‐kB signalling.^5^ Reports by Juozas Kupcinskas et al. revealed that the RNF7 gene variant confers an increased risk of developing liver fibrosis and cirrhosis.^6^ The roles of RNF7, however, in glioma have not been fully elucidated. Here, knockdown and overexpression experiments in vitro and in vivo indicated that RNF7 promotes glioma cell proliferation by facilitating cell cycle progression and inhibiting apoptosis. Cignal Finder Cancer 10‐Pathway Reporter Kits were adopted to screen for signalling pathways that might be involved in this process. The final results showed that the PI3K/AKT signalling axis was obviously inhibited, but the other signalling axis were not notably affected by RNF7 knockdown in T98G and U‐87MG cells. In addition, GSEA demonstrated that RNF7 was significantly associated with the PI3K/AKT signalling pathway. The above finding was further confirmed by rescue experiments. Thus, building on the above results, we report that RNF7 promotes tumour development by activating the PI3K/AKT signalling pathway.

## 2 MATERIALS AND METHODS

### 2.1 Reagents and cell lines

Normal human brain glial HEB cells, multiple human glioma cell lines U‐251, U‐87MG (glioblastoma of unknown origin), SHG44 and T98G were cultivated as reported in our previous work.^7^, ^8^ LY294002 (2‐morpholino‐8‐phenyl‐4H‐chromen‐4‐one) was purchased from Sigma‐Aldrich Co. (St. Louis, MO). Primary antibodies were used to detect RNF7 (ab181986; Abcam), AKT (ab179463; Abcam), p‐AKT (ab192623; Abcam), PI3K (ab191606; Abcam) and p‐PI3K (ab182651; Abcam). For more detailed information, please refer to our previous article.^9^, ^10^, ^11^

### 2.2 Glioma samples

A total of 57 glioma specimens and eight normal human brain samples (NBT) were obtained from the Union Hospital (Wuhan, Hubei, China) from April 2017 to August 2020 after obtaining informed consent from all participants and approval from the relevant research ethics committees (S182). None of the patients in this study received adjuvant, neoadjuvant or radiotherapy before surgery. All clinical samples and clinical information were gathered and handled in line with relevant guidelines and regulations.

### 2.3 Plasmid construction and lentivirus

First, the construction of overexpression plasmid pLVX‐Puro‐RNF7. Then, T98G and U‐87MG cells were treated with the corresponding expression plasmid. Detailed procedure is recorded in our previous study.^7^, ^8^, ^10^ Briefly, T98G and U‐87MG cells were routinely grown to 90% confluency in six‐well cell culture plates and transfected according to the manufacturer's instructions. Forty‐eight hours after infection, cells were selected with puromycin (2 μg/ml; Sigma‐Aldrich, #P8833) for 5–6 days to remove uninfected cells. Three short‐interfering RNAs (siRNAs) were designed according to the RNF7 sequence. We picked the two siRNAs with the greatest knockout effect.

### 2.4 CCK‐8 assay

Cell proliferation assay was performed with CCK‐8 (Cell Counting Kit‐8; Dojindo) according to the manufacturer's protocol. Cells were seeded in a 96‐well plate (6 × 10^4^ cells per well) with the appropriate fresh medium. Cell proliferation was examined after treatment with CCK‐8 solution, and the absorption rate was measured with a microplate reader (Bio‐Rad Laboratories). For more details, please refer to our previous publication.^9^, ^11^, ^12^

### 2.5 Western blotting

Tissues and cells were lysed with RIPA buffer and quantified using BCA (Beyotime). Subsequently, the lysates were subjected to SDS–PAGE and then transferred to polyvinylidene fluoride (PVDF) membrane. Then, PVDF membrane was incubated with corresponding antibodies under the right conditions and then subjected to a colour reaction. Please refer to ‘Cell lines and reagents’ for primary antibody information. For more details, please refer to our previous publication.^8^, ^12^, ^13^

### 2.6 Apoptosis assay

Cell apoptosis was analysed by PI/Annexin V‐FITC double assay and flow cytometry. A PE Annexin V Apoptosis Detection Kit I (Beyotime) was used in the light of reagent instructions. Briefly, all cells were collected and resuspended in the binding buffer. Subsequently, the cells were treated with propidium iodide (PI) and Annexin V‐FITC. Stained cells were analysed by flow cytometry, and the data were analysed by FlowJo software (FlowJo LLC).

### 2.7 qRT‐PCR

Total RNA extraction, cDNA synthesis and qRT‐PCR were performed as described before.^7^, ^8^ Relative mRNA expression levels were calculated relative to GAPDH.

### 2.8 Immunohistochemistry (IHC) and Immunofluorescence (IF)

The details of IHC and IF can refer to our previous study.^10^, ^11^ In summary, tissue was then dehydrated, paraffin‐embedded and sectioned. Subsequently, sections were then incubated with appropriate primary and secondary antibody. Immunohistochemistry scores were conducted referred to our previous article.^8^, ^12^, ^13^

### 2.9 Brain orthotopic xenografts

Orthotopic brain xenografting was generated as previously described.^7^, ^8^ Briefly, 6‐week‐old female BALB/c nude mice were divided randomly into the corresponding group, and each group had three mice. Animals were placed in a stereotaxic instrument (Stoelting Digital Mouse Stereotaxic Instrument), and their skull was exposed. The microsyringe (10 μl) was positioned with a stereotaxic instrument. Tumour cells exposed to different treatments were injected into the mouse brains by using a micropipette mounted on a stereotaxic instrument. Animals were imaged using the IVIS Lumina III in vivo imaging system (PerkinElmer). For more detail, please refer to our previous publication.^8^, ^12^, ^13^

### 2.10 Statistical methods and calculations

Statistical analysis was conducted with SPSS 21.0 software. Unpaired and paired t‐tests were used to compare two datasets, and one‐way anova was used to compare three datasets or more. Statistical significance was defined as a *p* < 0.05, and data are presented as the mean ± SD.

## 3 RESULTS

### 3.1 RNF7 was overexpressed in glioma and negatively associated with prognosis

**FIGURE 1:**
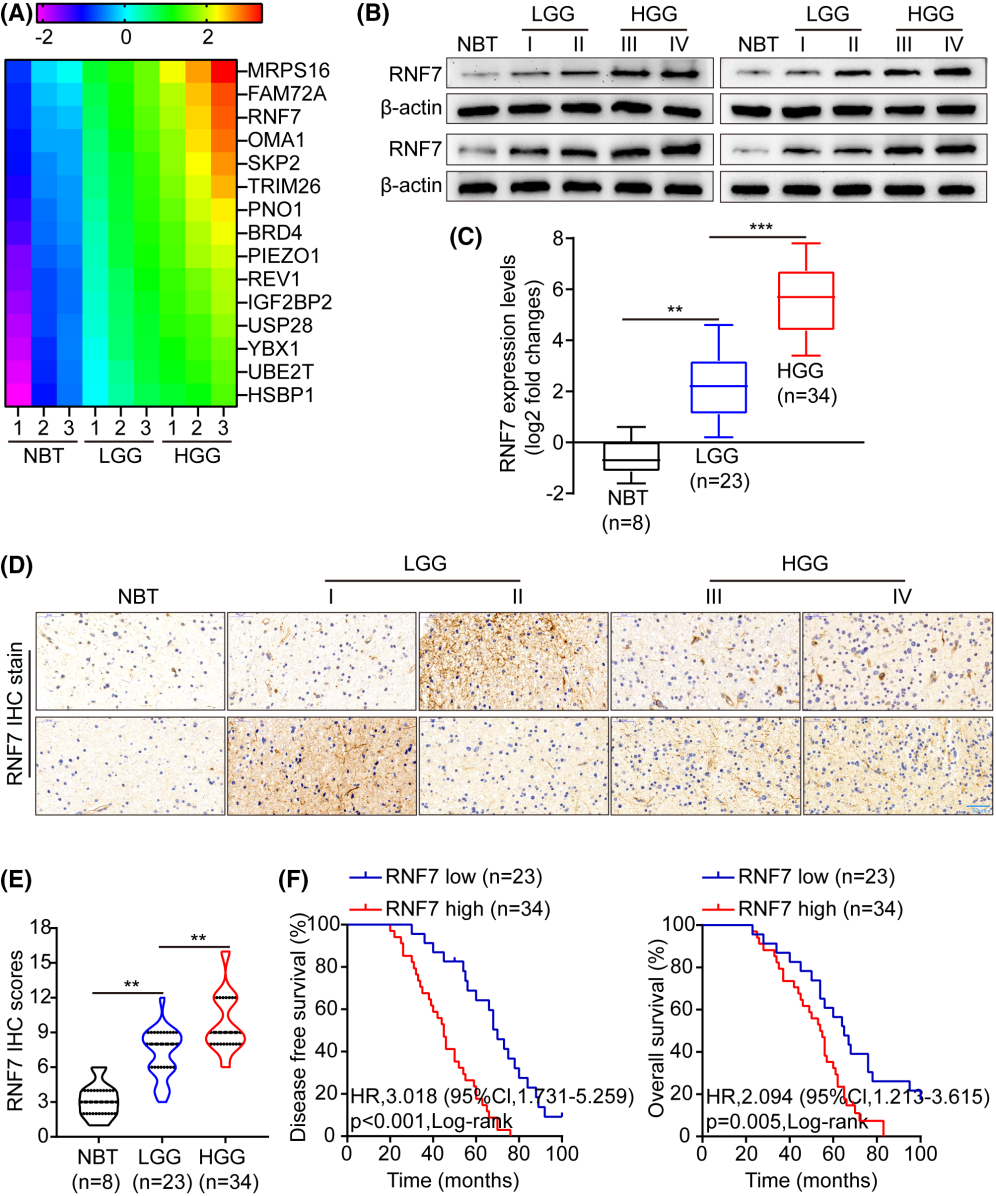
RNF7 was overexpressed in glioma and negatively associated with prognosis. (A) Heat map shows the top upregulated genes in different groups. (B) WB was used to detect the expression of RNF7 protein in tumour and normal tissues. (C) qRT‐PCR was used to detect RNF7 mRNA levels in tumour tissues and normal tissues. (D, E) IHC staining and scoring were used to detect the protein levels of RNF7 in tumour tissues and normal tissues. (F) Log‐rank test of DFS or OS was conducted. Data were presented as mean ± SD from three independent experiments. **p < 0.01 and ***p < 0.001

High‐throughput sequencing was used to screen candidate genes that lead to tumour progression (HGG vs. LGG, HGG: high‐grade glioma; LGG: low‐rade glioma). The top 15 upregulated genes are shown in Figure 1A, all of which were altered more than fivefold in HGG compared with LGG. Among them, RNF7 was significantly upregulated. To verify the differential expression of RNF7 in glioma, Western blotting (WB) and qRT‐PCR were used to measure RNF7 levels in normal brain tissues (NBT) and tumour tissues. Compared with NBT, RNF7 was significantly highly expressed in tumour tissues and was elevated more in HGG than in LGG (Figure 1B,C). Then, immunohistochemistry (IHC) was used to detect RNF7 protein levels in NBT and glioma tissues. Typical micrographs of IHC staining are shown in Figure 1D, which showed that compared with NBT, the immunohistochemical staining intensity of RNF7 was significantly enhanced in glioma tissues, especially HGG. In addition, there was a positive correlation between the IHC staining score and the grade of malignancy (Figure 1E). Besides, according to Kaplan–Meier analysis, patients with high expression of RNF7 had a poorer prognosis (Figure 1F and Figure S1A).

In order to assess the pathological and clinical predictive value of RNF7, the ROC analysis was conducted between RNF7‐based, WHO‐based and a combination of both to predict clinical outcomes. As measured by the area under the curve (AUC), the combination model (0.732) outperformed the WHO‐based model alone (0.604). It seems that RNF7 combined with WHO stage could better predict clinical outcome than WHO stage alone (Figure S1B,C). In addition, we examined the correlation between RNF7 mRNA levels and clinicopathological characteristics in 57 glioma specimens. Table 1 shows the clinical, pathological and tumour molecular features according to the RNF7 mRNA expression level. The results showed that the RNF7 mRNA expression level was highly associated with the Karnofsky Performance Scale (KPS) score (*p* = 0.026), tumour size (*p* = 0.0003), tumour grade (*p* = 0.027) and recurrence (*p* = 0.002). Further univariate and multivariate Cox regression analyses indicated that a high RNF7 mRNA expression level was an independent prognostic factor for poor survival of patients with glioma (Table 2). Table 2 shows that a high RNF7 mRNA expression level is correlated with tumour grade and tumour recurrence. Together, these results indicate that RNF7 expression is positively associated with clinical glioma malignant grade and negatively associated with the prognosis of patients.

**TABLE 1:** Association of RNF7 expression with clinicopathological characteristics in human gliomas

Features	No.	RNF7		*p*‐Value
		Low	High	
Age(years)				
<50	27	13	14	0.915
> = 50	30	14	16	
Gender				
Male	25	15	10	0.087
Female	32	12	20	
Tumour size, cm				
<2	35	22	13	0.003
> = 2	22	5	17	
Tumour location				
Supratentorial	31	14	17	0.709
Subtentorial	26	13	13	
Karnofsky Performance Scale				
<90	30	10	20	0.026
> = 90	27	17	10	
WHO grade				
Low‐grade(I + II)	23	15	8	0.027
High‐grade(III + IV)	34	12	22	
Tumour recurrence				
No	30	20	10	0.002
Yes	25^a^	6^a^	19^a^	

^a^ Some data are not available; statistics are based on available data.

**TABLE 2:** Single factor and multiple factor analysis table

	Univariate			Multivariate		
	*p*	HR	95% CI	*p*	HR	95% CI
RNF7	0.018	1.572	1.182–2.428	<0.001	1.738	1.243–4.214
Tumour size, cm	<0.001	1.462	1.252–2.893	0.025	2.142	1.473–3.792
Karnofsky Performance Scale	0.042	1.693	1.372–2.792	<0.001	1.387	1.213–2.826
WHO grade	<0.001	1.753	1.354–3.571	<0.001	1.631	1.361–2.792
Tumour recurrence	<0.001	2.142	1.853–4.262	<0.001	1.936	1.452–3.629

Abbreviation: CI, confidence interval.

### 3.2 Overexpression of RNF7 facilitates cell growth and cell cycle progression and inhibits apoptosis

**FIGURE 2:**
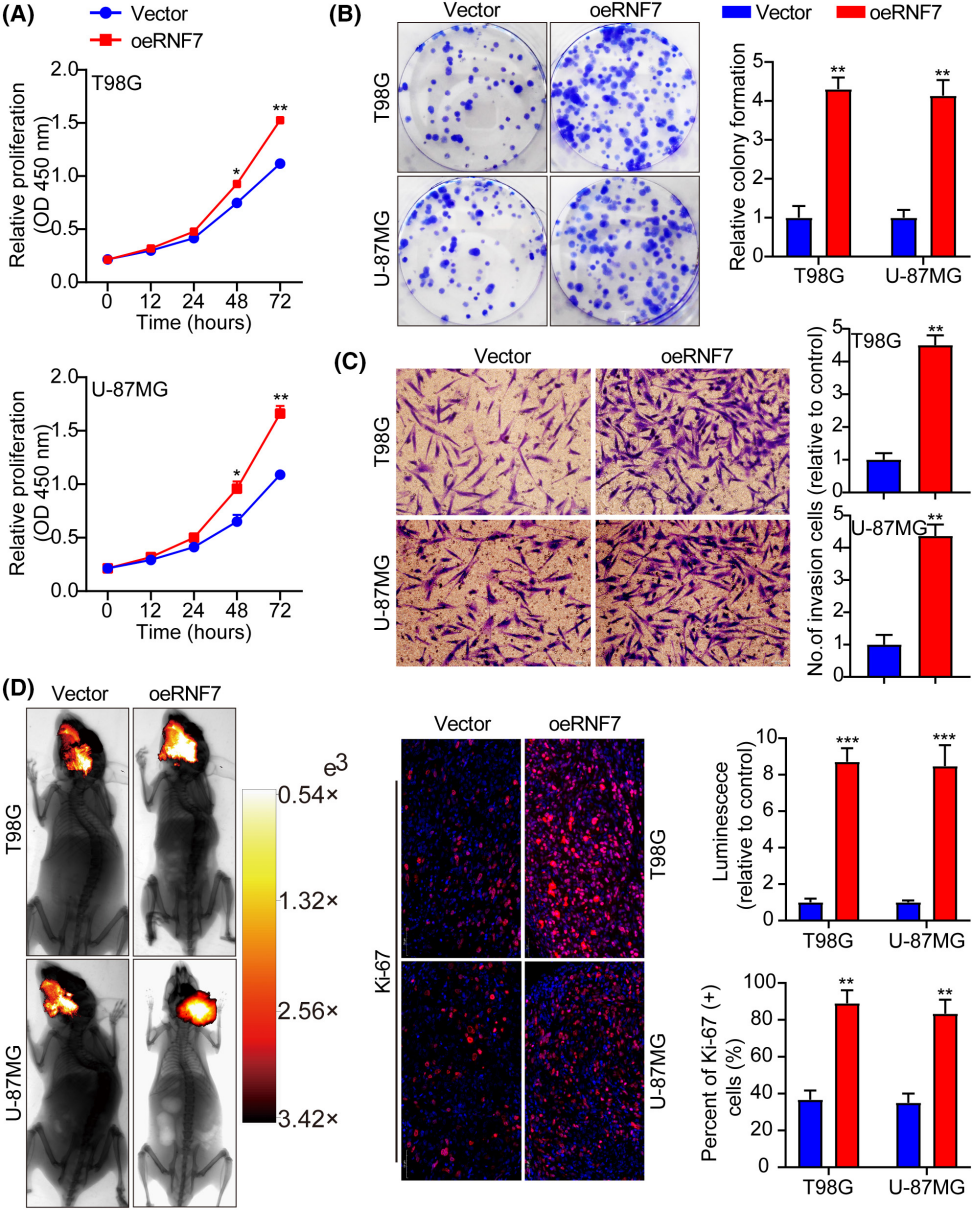
Overexpression of RNF7 facilitates cell cycle progression and cell growth and inhibits apoptosis. (A) Cell growth curves measured by CCK‐8 between Vector and oeRNF7. (B) RNF7 overexpression facilitated colony formation and histogram quantification (panels). (C) Invasion assays showing that overexpression of RNF7 facilitates cell invasion. The numbers of invading cells are shown. Bars: 50 μm. (D) Typical pictures of live animal imaging between Vector and oeRNF7. Representative pictures of Ki‐67 staining between Vector and oeRNF7. Data were presented as mean ± SD from three independent experiments. *p < 0.05, **p < 0.01 and ***p < 0.001.

To determine the function of RNF7 in glioma progression, the expression of RNF7 in multiple human glioma cell lines, U‐251, U‐87MG, SHG44, T98G and HEB normal human brain glial cells was detected, and RNF7 was highly expressed in glioma cells (Figure S1D). Next, overexpressed RNF7 in T98G and U‐87MG cells was verified using WB (Figure S1E). RNF7 overexpression facilitated cell proliferation and inhibited apoptosis in T98G and U‐87MG cells (Figure 2A,B and Figure S2A). Transwell experiment showed that RNF7 overexpression promoted tumour cell invasion (Figure 2C). In addition, cell cycle distribution indicated that RNF7 overexpression promoted G1 to S‐phase cell cycle progression and accelerated cell proliferation (Figures S2B and S3A). Animal studies also confirmed that RNF7 overexpression significantly facilitated tumour growth in vivo (Figure 2D). Ki‐67 immunohistochemical staining was performed to assess the proliferative index of the tumour, which also indicated that proliferation in the RNF7 overexpression group was higher than that of the control group (Figure 2D). These data show that RNF7 overexpression can promote cell proliferation and G1/S cell cycle progression and inhibit cell apoptosis.

### 3.3 Knockdown of RNF7 suppresses cell growth and cell cycle progression and promotes apoptosis

**FIGURE 3:**
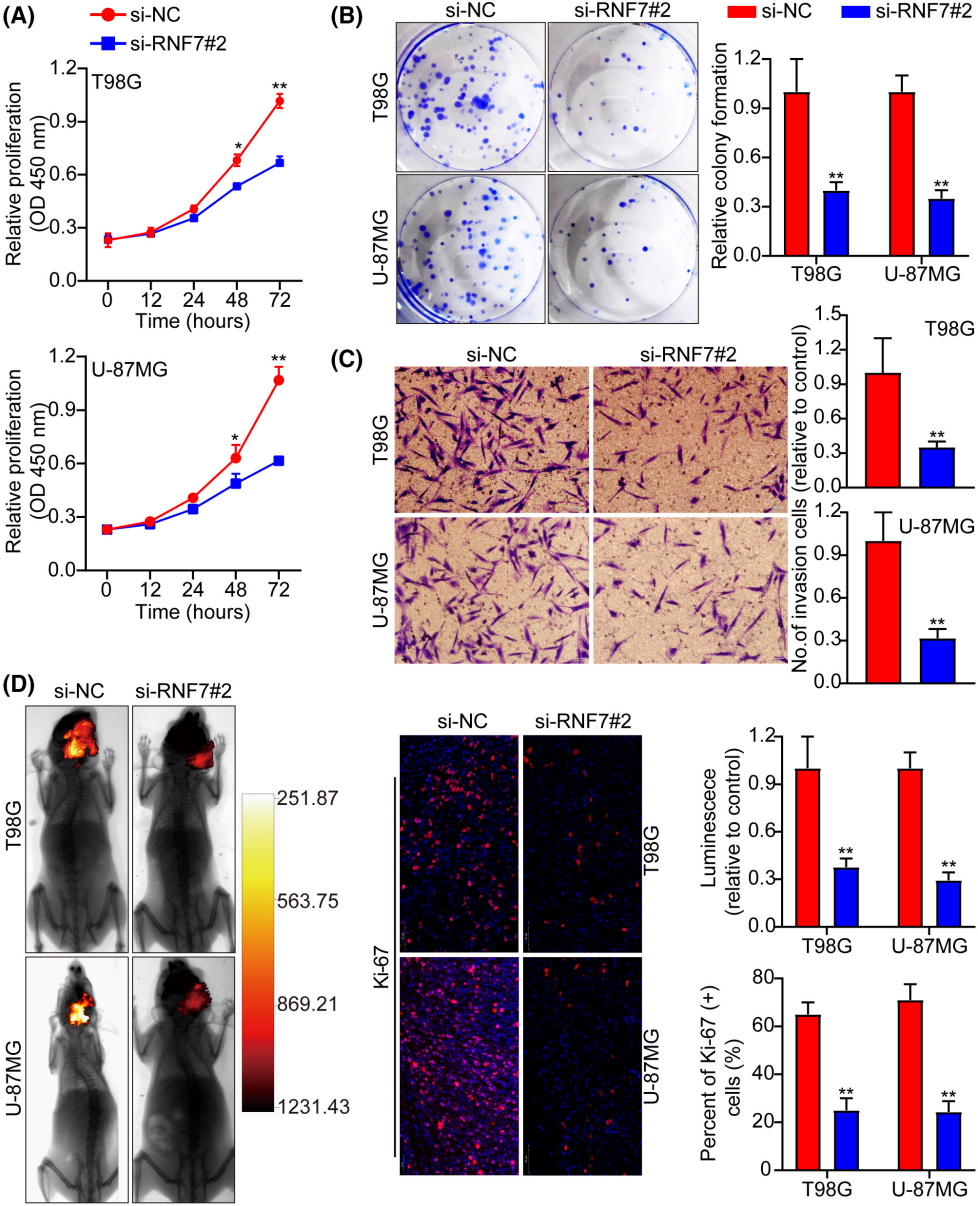
Knockdown of RNF7 suppresses cell cycle progression and cell growth and promotes apoptosis. (A) Cell growth curves measured by CCK‐8 assay between si‐NC and siRNF7#2. (B) RNF7 overexpression inhibited colony formation and histogram quantification (panels). (C) Invasion assays showing that RNF7 knockdown inhibits cell invasion. The numbers of invading cells are shown. Bars: 50 μm. (D) Typical pictures of live animal imaging between si‐NC and siRNF7#2. Representative pictures of Ki‐67 staining between si‐NC and siRNF7#2. Data were presented as mean ± SD from three independent experiments. *p < 0.05 and **p < 0.01

Next, RNF7 was knocked down in T98G and U‐87MG cells and the knockdown efficiency was verified using WB (Figure S1E). RNF7 knockdown suppressed cell proliferation and promoted apoptosis in T98G and U‐87MG cells (Figure 3A,B and Figures S3B and S7B). Transwell experiment showed that RNF7 knockdown inhibited tumour cell invasion (Figure 3C). In addition, cell cycle distribution indicated that inhibition of RNF7 induced cell cycle arrest at the G1 phase (Figure S4A). Animal studies also confirmed that RNF7 knockdown significantly inhibited tumour growth in vivo (Figure 3D). Ki‐67 immunohistochemical staining was used to assess the proliferative index of the tumour, which also indicated that proliferation in the RNF7 knockdown group was lower than that of the control group (Figure 3D). These data show that knockdown of RNF7 inhibits cell proliferation, suppresses tumour cell cycle progression by inducing G1 phase arrest and promotes glioma cell apoptosis.

### 3.4 RNF7 activates the PI3K/AKT signalling

**FIGURE 4:**
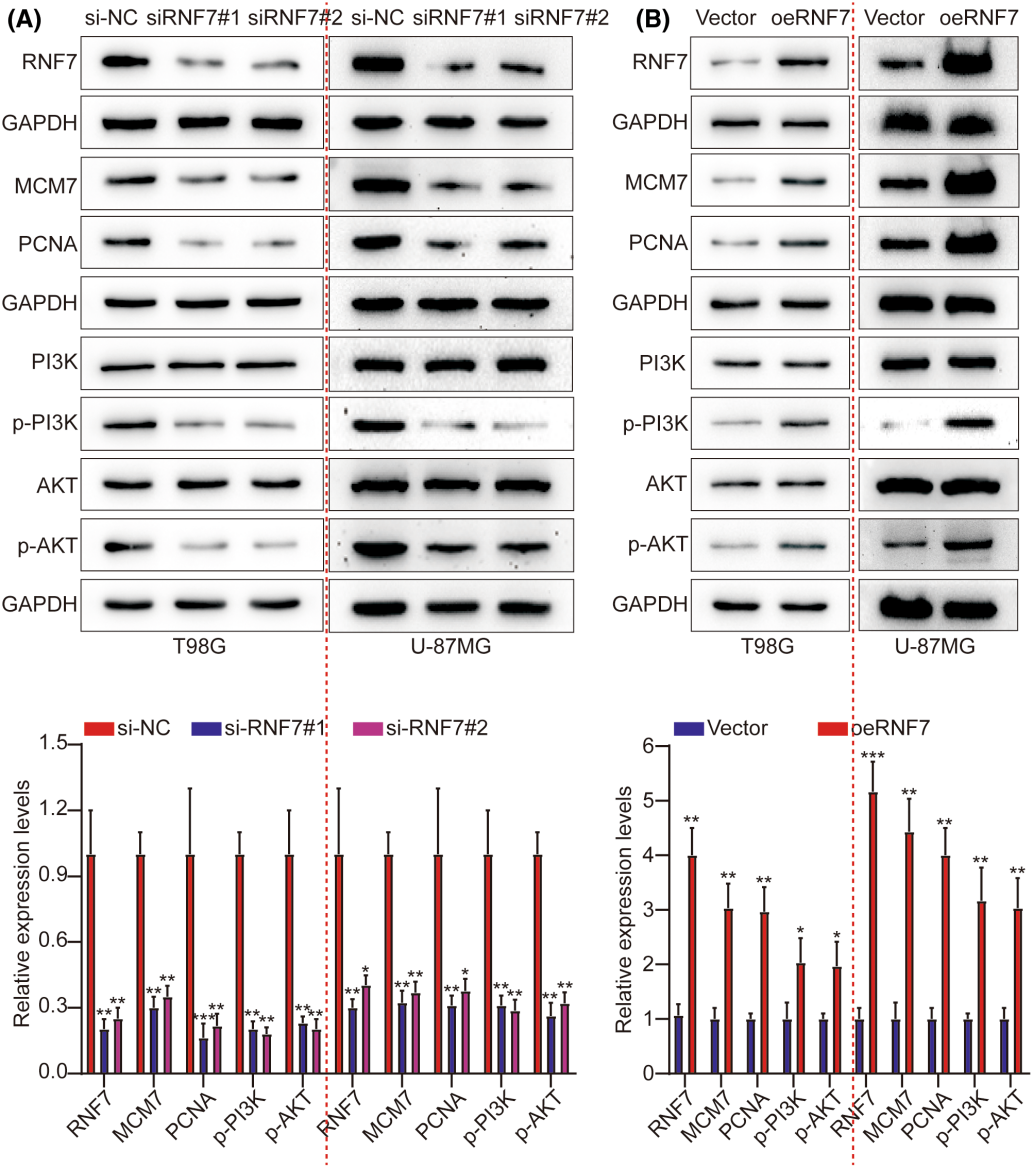
RNF7 activates the PI3K/AKT signalling and thereby drives tumour growth. (A) Expression of RNF7, MCM7, PCNA, AKT, p‐AKT, PI3K and p‐PI3K was detected by WB between si‐NC, siRNF7#1 and siRNF7#2. (B) Expression of RNF7, MCM7, PCNA, AKT, p‐AKT, PI3K and p‐PI3K was detected by WB between Vector and oeRNF7.

Next, we performed GSEA to screen the signalling pathway associated with RNF7. GSEA indicated that RNF7 was significantly associated with the PI3K/AKT signalling (Figure S5B). Besides, Cignal Finder Cancer 10‐Pathway Reporter Kits were adopted to screen for signalling pathways that might be involved in this process. The final results showed that the PI3K/AKT signalling axis was obviously inhibited, but the other signalling axis was not notably affected by RNF7 knockdown in T98G and U‐87MG cells (Figure S7A). To further validate whether RNF7 promotes tumour growth in tumour cells by activating the PI3K/AKT pathway, we knocked down and overexpressed human RNF7 in T98G and U‐87MG cells, respectively. Our results indicated that knockdown of RNF7 resulted in downregulation of the phosphorylation levels of PI3K (p‐PI3K) and AKT (p‐AKT) but not total PI3K and AKT, while the proliferation‐related proteins MCM7 and PCNA were also downregulated (Figure 4A). As expected, overexpression of RNF7 promoted p‐PI3K and p‐AKT but not PI3K and AKT, as well as the proliferation‐related proteins MCM7 and PCNA (Figure 4B). These data indicated that RNF7 activates the PI3K/AKT pathway.

### 3.5 RNF7 influences tumour growth via activating this PI3K/AKT signalling

**FIGURE 5:**
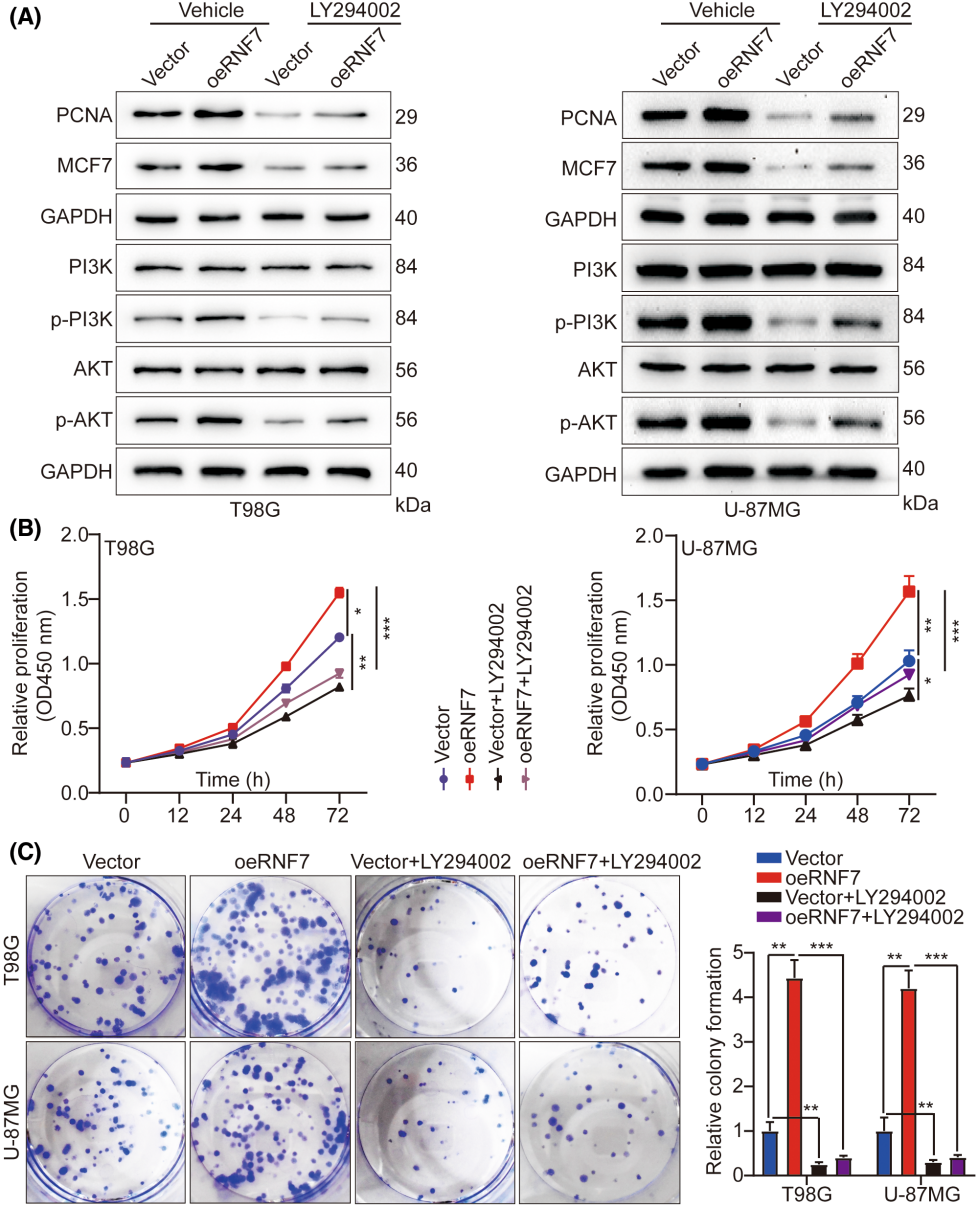
RNF7 influences tumour growth via activating the PI3K/AKT signalling. (A) Expression of RNF7, MCM7, PCNA, AKT, p‐AKT, PI3K and p‐PI3K was measured by WB in different treatment groups. (B) Cell growth curves measured by CCK‐8 assay in different treatment groups. (C) Cell growth measured by colony formation assay in different treatment groups and histogram quantification (panels). Data were presented as mean ± SD from three independent experiments. *p < 0.05, **p < 0.01 and ***p < 0.001

**FIGURE 6:**
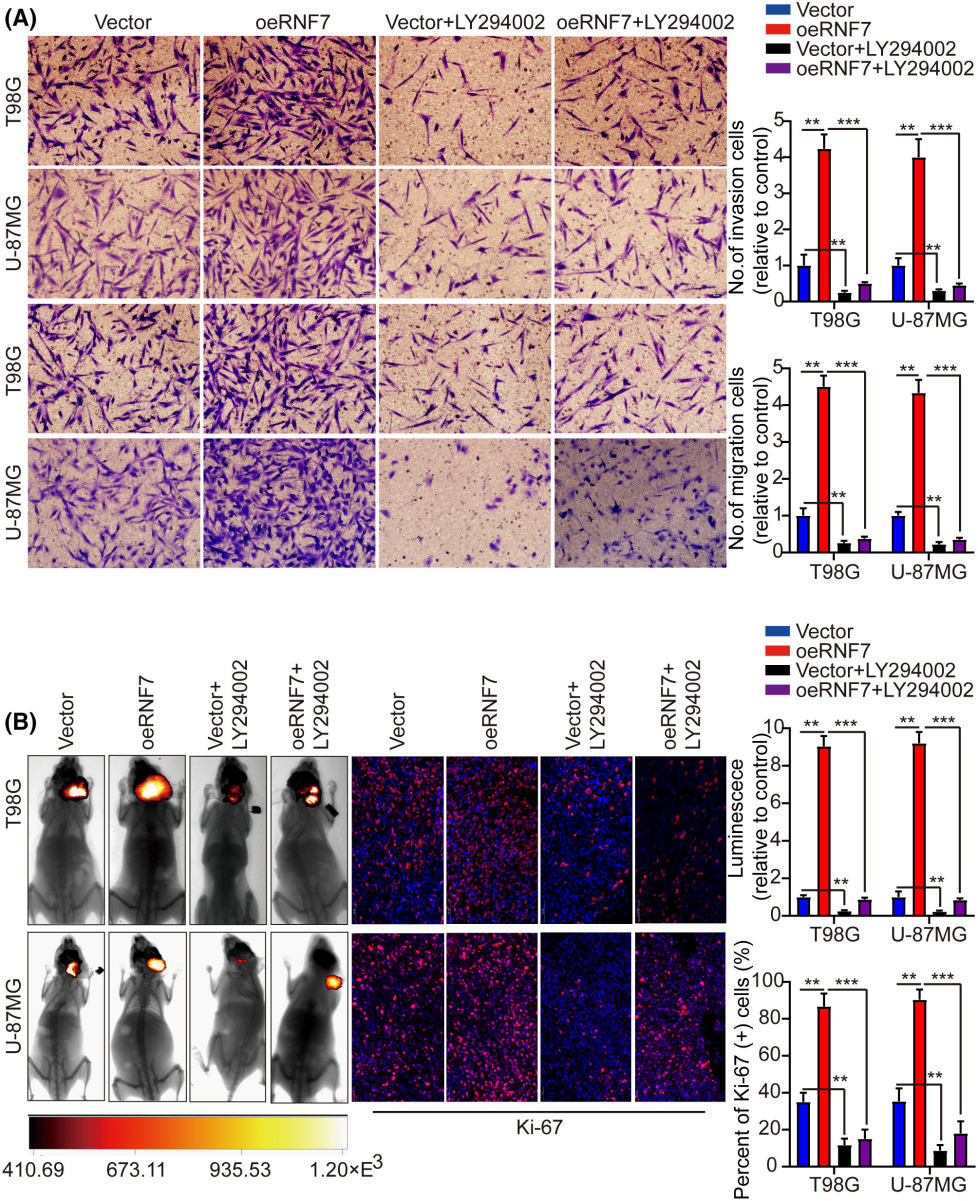
RNF7 influences tumour growth via activating the PI3K/AKT signalling. (A) RNF7 overexpression could promote tumour cell migration, invasion and the effect could be attenuated by treatment with PI3K/AKT signalling pathway inhibitors LY294002. (B) Typical pictures of live animal imaging in different treatment groups and representative pictures of Ki‐67 staining in different treatment groups. Data were presented as mean ± SD from three independent experiments. **p < 0.01 and ***p < 0.001

To further determine whether RNF7 promotes cell proliferation via activating this PI3K/AKT, we treated T98G and U‐87MG cells with PI3K/AKT inhibitor LY294002 and investigated the effect of RNF7 overexpression on proliferation. In agreement with our previous conclusion, RNF7 overexpression induced p‐PI3K and p‐AKT but not PI3K and AKT, whereas RNF7 overexpression upregulated the proliferation‐related indices of MCM7 and PCNA. However, this effect was weakened when T98G and U‐87MG cells were treated with PI3K/AKT inhibitor LY294002 (Figure 5A). Furthermore, the CCK‐8 and colony formation assay indicated that LY294002 significantly reversed the effect of RNF7 on promoting T98G and U‐87MG cell proliferation (Figure 5B,C). Moreover, LY294002 dramatically induced apoptosis in T98G and U‐87MG cells overexpressing RNF7 and blocked cell cycle progression (Figures S5A and S6A). Transwell experiment showed that LY294002 significantly reversed the effect of RNF7 on promoting T98G and U‐87MG cells migration, and invasion (Figure 6A). Consistent with the in vitro study, the in vivo data confirmed that LY294002 treatment remarkably reduced tumour growth in mice with orthotopic transplantation glioma compared with the corresponding group (Figure 6B). The decreased Ki‐67 expression in xenograft tumours of LY294002‐treated mice also suggested that inhibition of PI3K/AKT signalling could counteract the effect of RNF7 on promoting cell proliferation (Figure 6B). Taken together, RNF7 might promote glioma progression via activating this PI3K/AKT signalling.

## 4 DISCUSSION

Recently, the dysregulation of RNF7 and its biological function in many diseases have been frequently reported. However, the possible role of RNF7 in glioma has not been reported.

Here, we report the clinical relevance of RNF7 as a prognostic marker and the biological function of RNF7 in glioma. In this study, we found that RNF7 was overexpressed in human glioma tissue and cell lines and promoted glioma cell proliferation both in vitro and in vivo, indicating that RNF7 plays a critical role in glioma. Interestingly, RNF7 promoted tumour cell proliferation by inducing G1 to S cell cycle phase transition and inhibiting apoptosis. Mechanistically, overexpression of RNF7 may activate the PI3K/AKT signalling pathway to boost tumour progression.

Accumulating evidence indicates that RNF7 functions as an oncogene and plays a key role in transformation and tumour progression. Xiao et al.^14^ reported that RNF7 overexpression promotes prostate cancer tumorigenesis by activating the ERK1/2 signalling. Reports by Zheng et al.^15^ indicated that RNF7 is often overexpressed in patients with glioblastoma multiform (GBM) and significantly correlated with poor outcome. However, neither the function of RNF7 nor its mechanism were elucidated in glioma. Herein, our data indicated that RNF7 was highly expressed in glioma, and a high expression level of RNF7 conferred poor prognosis and could act as an independent prognostic marker for glioma. The loss/gain‐of‐function study revealed that RNF7 facilitated cell cycle progression and proliferation and suppressed apoptosis of glioma cells in vivo and in vitro. Cignal Finder Cancer 10‐Pathway Reporter Kits were adopted to screen for signalling pathways that might be involved in this process. The final results showed that the PI3K/AKT signalling axis was obviously inhibited, but the other signalling axis was not notably affected by RNF7 knockdown in T98G and U‐87MG cells. Besides, GSEA found that PI3K/AKT pathway might be related to the function of RNF7 in tumour, which was further verified by WB and rescue experiments using LY294002, a widely used PI3K/AKT inhibitor. This PI3K/AKT pathway, one of the most active pathways in tumours, has been reported to exert critical roles in malignancies.^16^, ^17^, ^18^ Longzhou Zhang et al.^19^ reported that in a PFKFB4‐dependent manner, E2F2 promotes glioma progression via PI3K/AKT. Xiaoming Zhang et al.^20^ reported that LPP‐AS2 regulates glioma tumorigenesis via miR‐7‐5p/EGFR/PI3K/AKT/c‐MYC feedback loops. Yanli Hou et al. reported that ACT001 targets glioma stem‐like cells by inhibiting AEBP1/PI3K/AKT signalling.^21^ Seyed H Shahcheraghi et al.^22^ reported that PI3K/AKT signalling pathway can be used as a drug target for glioma. These prior reports, alongside our own work, collectively indicate that this PI3K/AKT pathway promotes malignant progression in tumours. These prior reports, alongside our own work, collectively indicate that this PI3K/AKT pathway promotes malignant progression in tumours.

In summary, our results show that RNF7 plays a key biological role in human glioma by promoting proliferation and cell cycle progression and suppressing apoptosis of glioma cells by this PI3K/AKT pathway. Retrospective clinical data have revealed that RNF7 may provide a novel predictive marker of patient prognosis. In addition, from a therapeutic perspective, targeting the RNF7/PI3K/AKT axis may act as a new perspective on the prevention or treatment of glioma.

## AUTHOR CONTRIBUTIONS

**Nan Tang:** Conceptualization (equal); data curation (equal); formal analysis (equal); investigation (equal); methodology (lead); project administration (equal); resources (equal); software (equal); supervision (equal); validation (equal); writing – original draft (equal). **Kai Zhu:** Conceptualization (supporting); data curation (equal); formal analysis (supporting); investigation (supporting); methodology (supporting); software (supporting); validation (supporting). **Cheng Jiang:** Conceptualization (supporting); data curation (supporting); formal analysis (supporting); investigation (supporting); methodology (supporting); software (supporting); supervision (supporting); validation (supporting); visualization (supporting). **Zhi yong Xiong:** Conceptualization (supporting); data curation (supporting); formal analysis (supporting); funding acquisition (supporting); investigation (supporting); methodology (supporting); validation (supporting). **Qiang‐Ping Wang:** Conceptualization (supporting); data curation (supporting); formal analysis (supporting); funding acquisition (supporting); investigation (supporting); methodology (supporting); resources (supporting); supervision (supporting); validation (supporting); visualization (supporting). **Jun jun Li:** Conceptualization (equal); data curation (equal); formal analysis (equal); funding acquisition (lead); methodology (equal); project administration (lead); software (equal); supervision (equal); validation (equal); writing – original draft (equal); writing – review and editing (equal). **Xu wei ming:** Conceptualization (equal); data curation (equal); formal analysis (equal); funding acquisition (equal); investigation (equal); methodology (equal); project administration (equal); resources (lead); supervision (equal); writing – original draft (equal); writing – review and editing (equal).

## CONFLICT OF INTEREST

The authors declare that they have no competing interests.

## Supporting information


Figure S1–S7

## Data Availability

The datasets used during the present study are available from the corresponding author upon reasonable request.
