# Correction to “RNF7 promotes glioma growth via the PI3K/AKT signalling axis”

**DOI:** 10.1111/jcmm.70148

**Published:** 2024-11-25

Tang N, Zhu K, Jiang C, et al. RNF7 promotes glioma growth via the PI3K/AKT signalling axis. *J Cell Mol Med* 2023;27(2):277–286. doi: 10.1111/jcmm.17656.

In Tang et al. in the published version of the article Figure 2C, the two pictures of U‐87MG in the transwell invasion experiment were misquoted, and in Figure 5A, the two pictures of U‐87MG in the WB experiment, p‐PI3K and p‐AKT, are misquoted and come from the same picture. And in Figure 6A, the picture of T98G in the transwell invasion experiment were misquoted. The correct figures are shown below.

**FIGURE 2:**
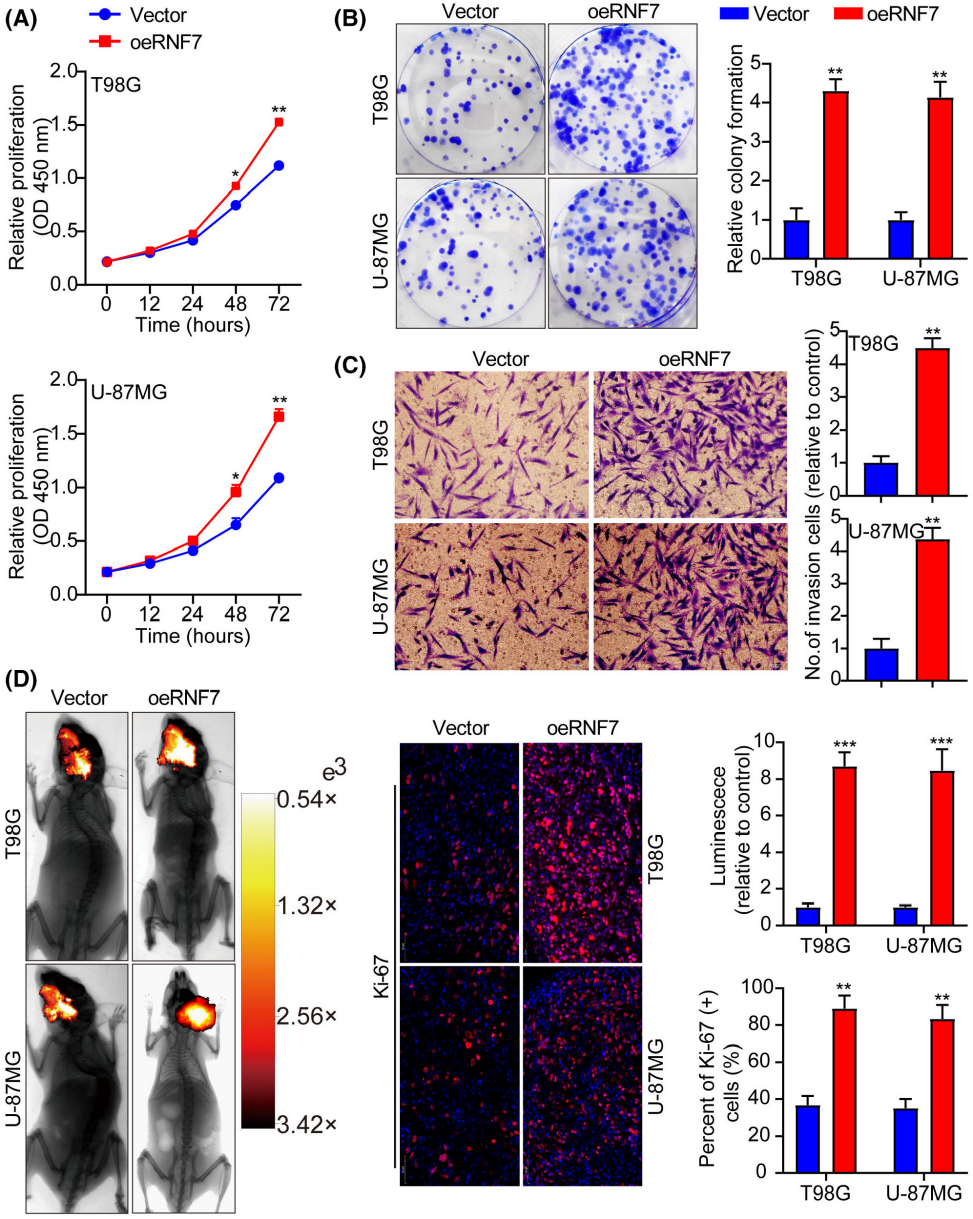
We confirm that there is no problem with the corrected image.

**FIGURE 5:**
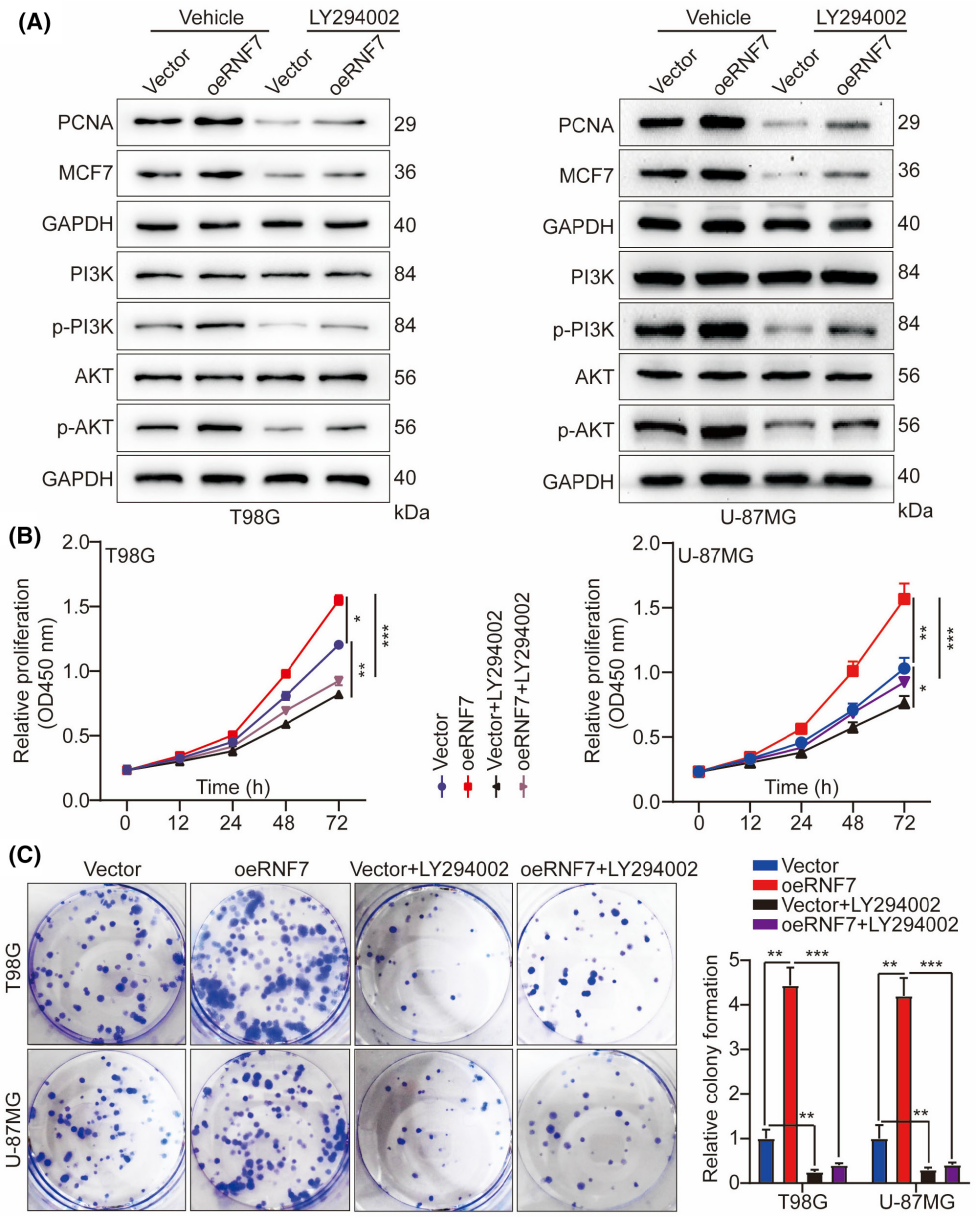
We confirm that there is no problem with the corrected image.

**FIGURE 6:**
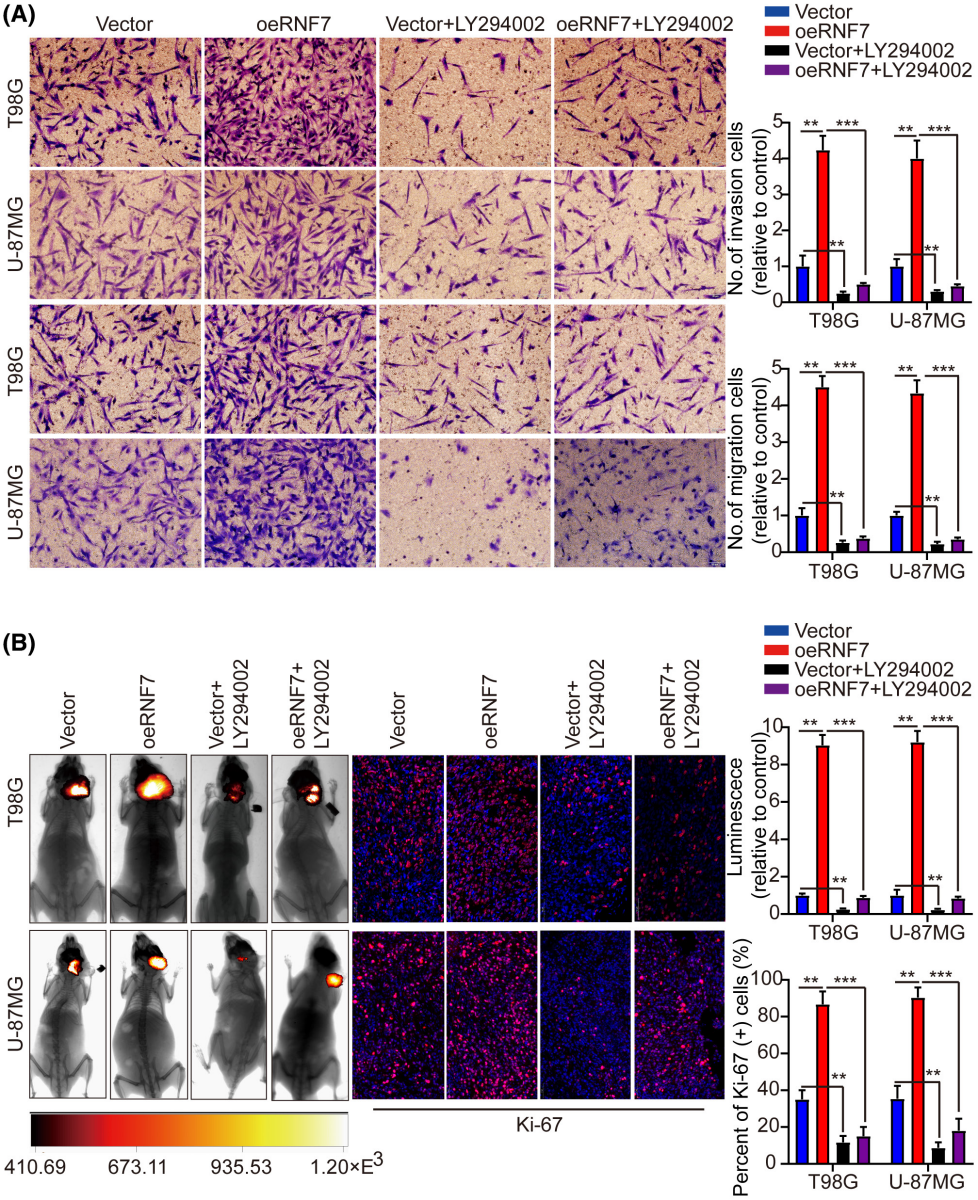
We confirm that there is no problem with the corrected image.

