# Supplementary material for: RNF7 promotes glioma growth via the PI3K/AKT signalling axis
Source: J Cell Mol Med. 2022 Dec 28;27(2):277–86. doi: 10.1111/jcmm.17656 (PMC9843527; doi:10.1111/jcmm.17656)
Supplement: Supplementary file 1 — Figure S1–S7 [file JCMM-27-277-s001.docx]

**RNF7 promotes glioma growth via the PI3K/AKT signaling axis**

Nan Tang^1^, Kai Zhu^1^, Cheng Jiang^1^, Zhiyong Xiong^1^, Qiangping Wang^1^, Junjun Li^1*^, Weiming Xu^1*^

Materials And Methods

Cignal Finder Cancer 10-Pathway Reporter Array Pathway analyses were conducted with the Cignal Finder Cancer 10-Pathway Reporter Array (QIAGEN, Germany) according to the manufacturer’s instructions. The suspended cells (6×10^3^/mL, 50 μL/well) were seeded into 96-well plates containing luciferase reporters for common targets in cancer pathways. Then, the cells were incubated at 37°C and 5% CO2 for 24 h, and luciferase activity analyses were performed using the Dual-Luciferase Reporter Kit (Promega, USA).

**Supplementary Figure Legends**


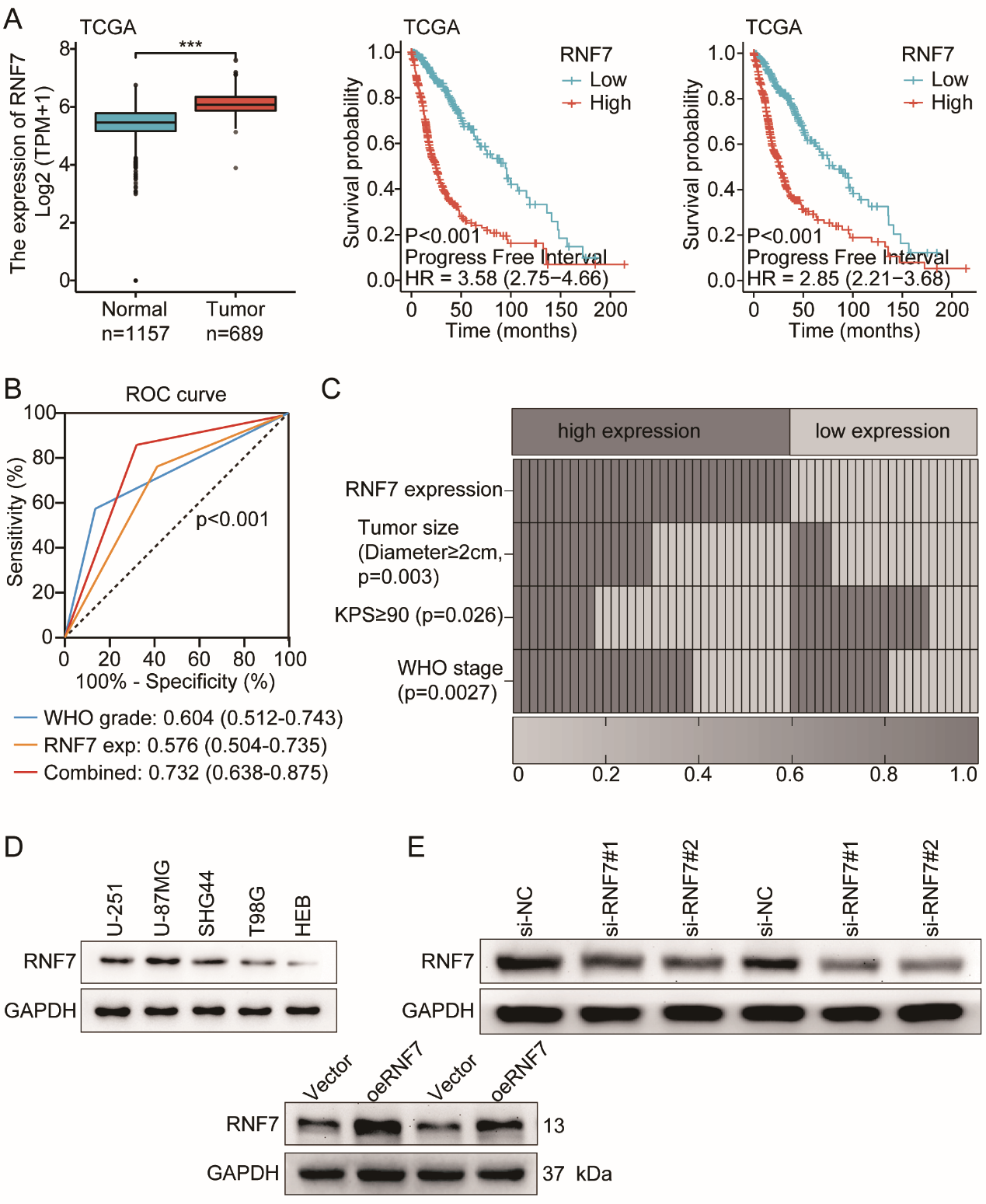


**Fig. S1 A** The results of the TCGA database showed that RNF7 was highly expressed in tumor tissues, and the expression level was inversely proportional to the prognosis of patients**.** **B** ROC analysis of RNF7-based, WHO-based and the combination model in predicting clinical outcome. **C** The heatmap illustrates the association of different clinical characters with RNF7 high and low-expression tumors. **D** WB to detect the expression levels of RNF7 between HEB, U-251, U-87MG, SHG44 and T98G cells. **E** Validation of knockdown and overexpression efficiency of RNF7 by WB. Data shown are mean ± SD (n = 3). (***P < 0.001).


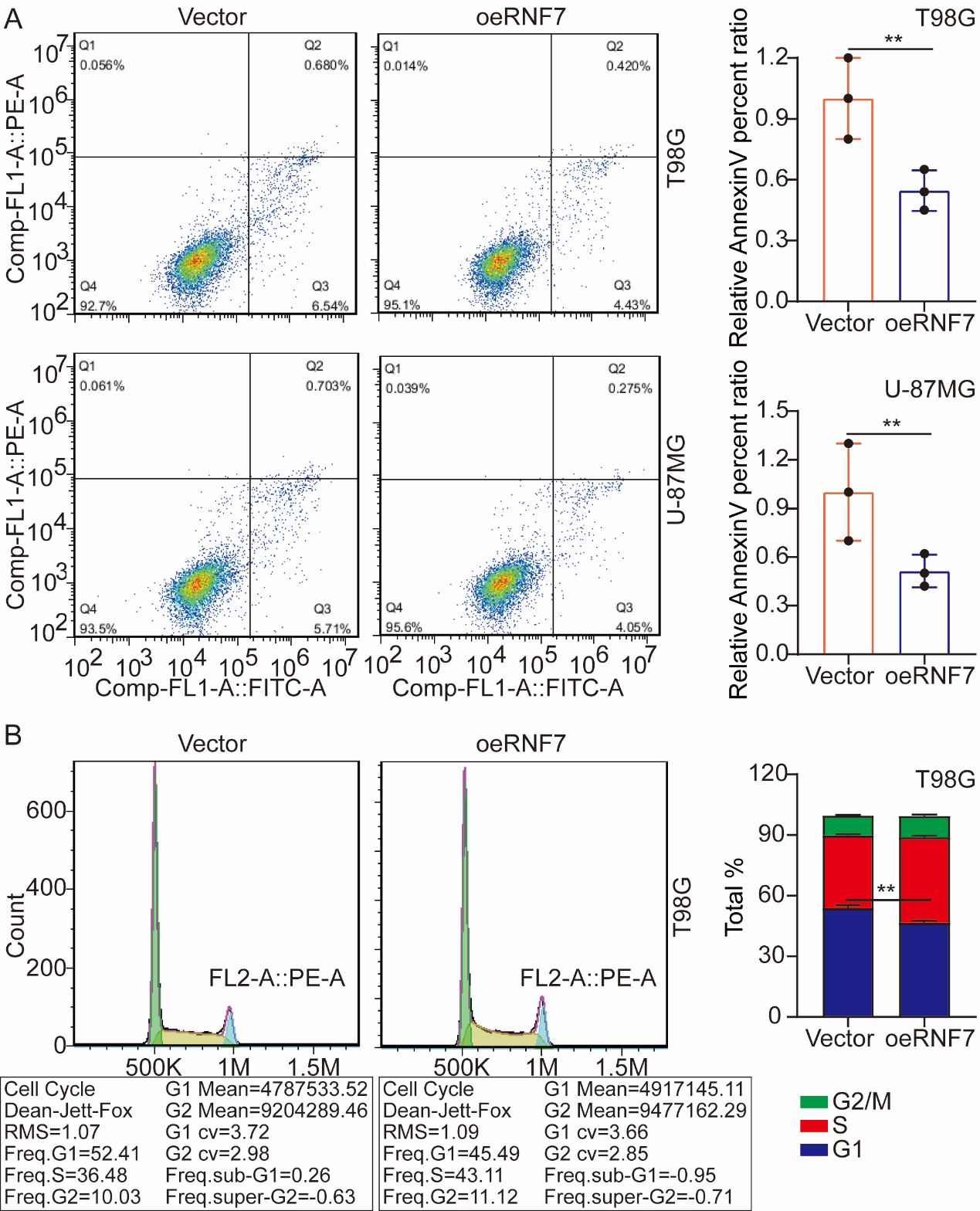


**Fig. S2 A.** The percentages of cell death, late apoptosis, early apoptosis, and live cells were calculated between Vector and oeRNF7. The figure on the right represents a column chart. **B.** RNF7 overexpression promoted cell cycle progression. The figure on the right represents a column chart.


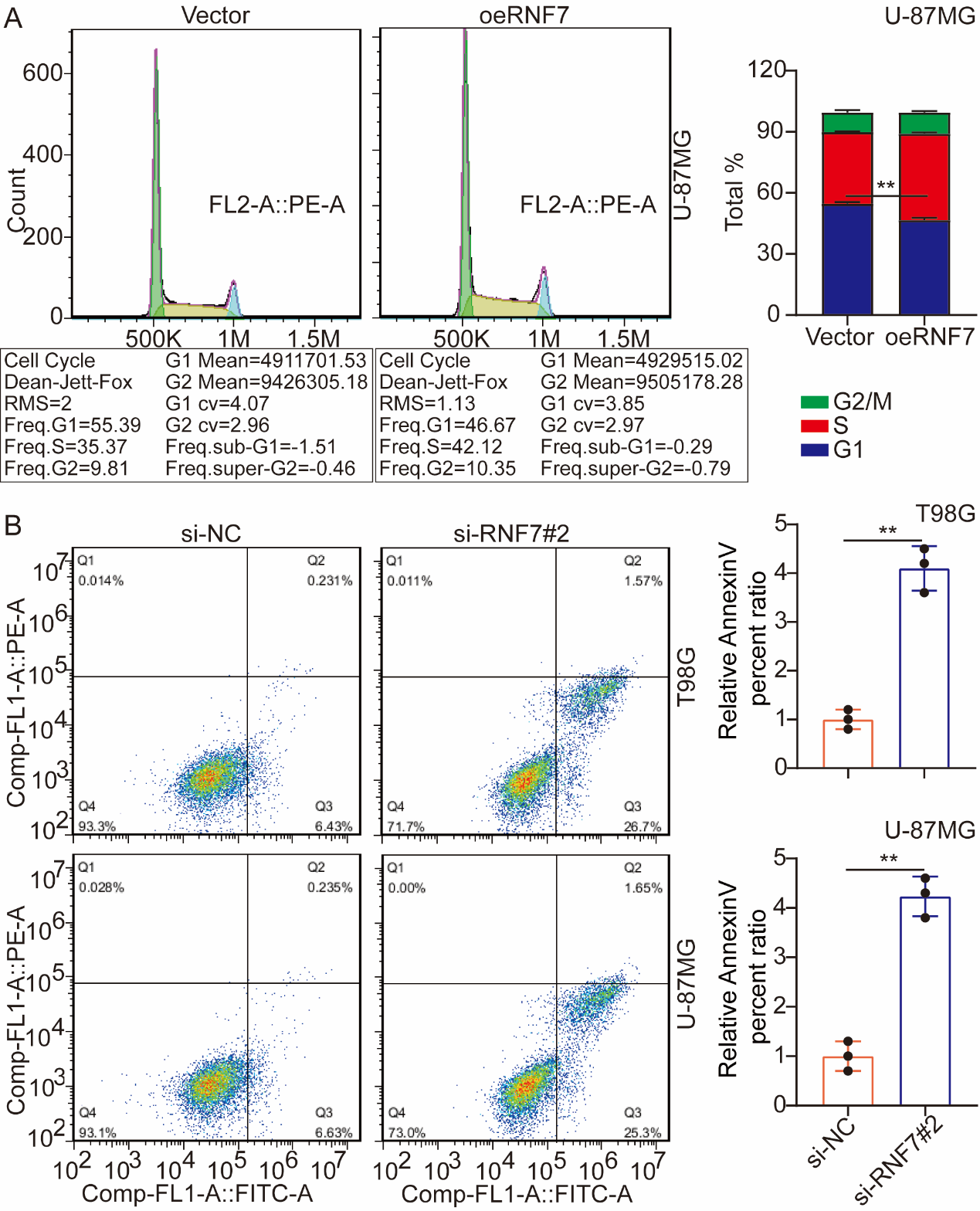


**Fig. S3 A.** RNF7 overexpression promoted cell cycle progression. The figure on the right represents a column chart. **B.** The percentages of cell death, late apoptosis, early apoptosis, and live cells were calculated between si-NC and siRNF7#2. The figure on the right represents a column chart.


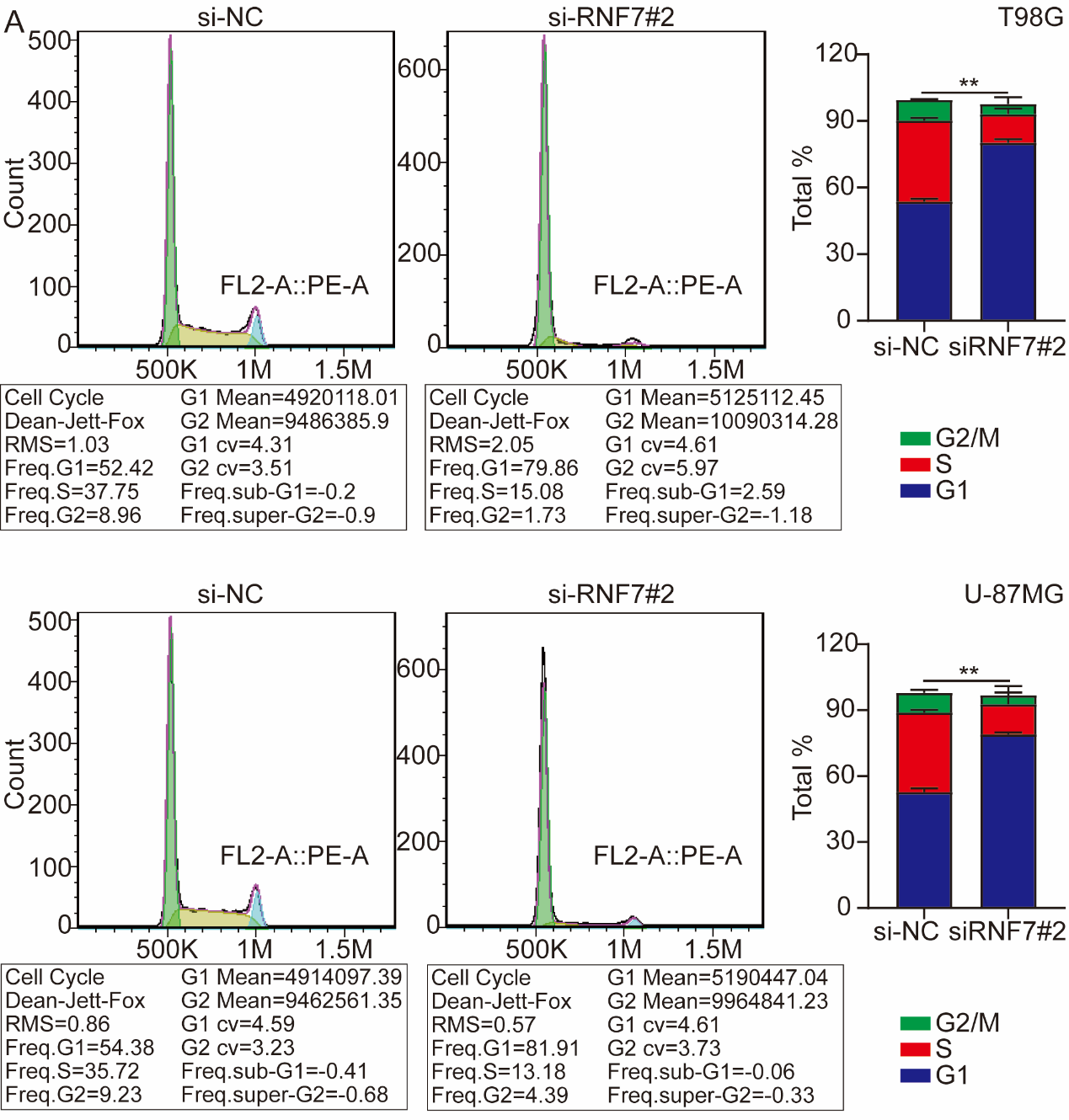


**Fig. S4 A.** RNF7 knockdown blocked cell cycle progression. The figure on the right represents a column chart.


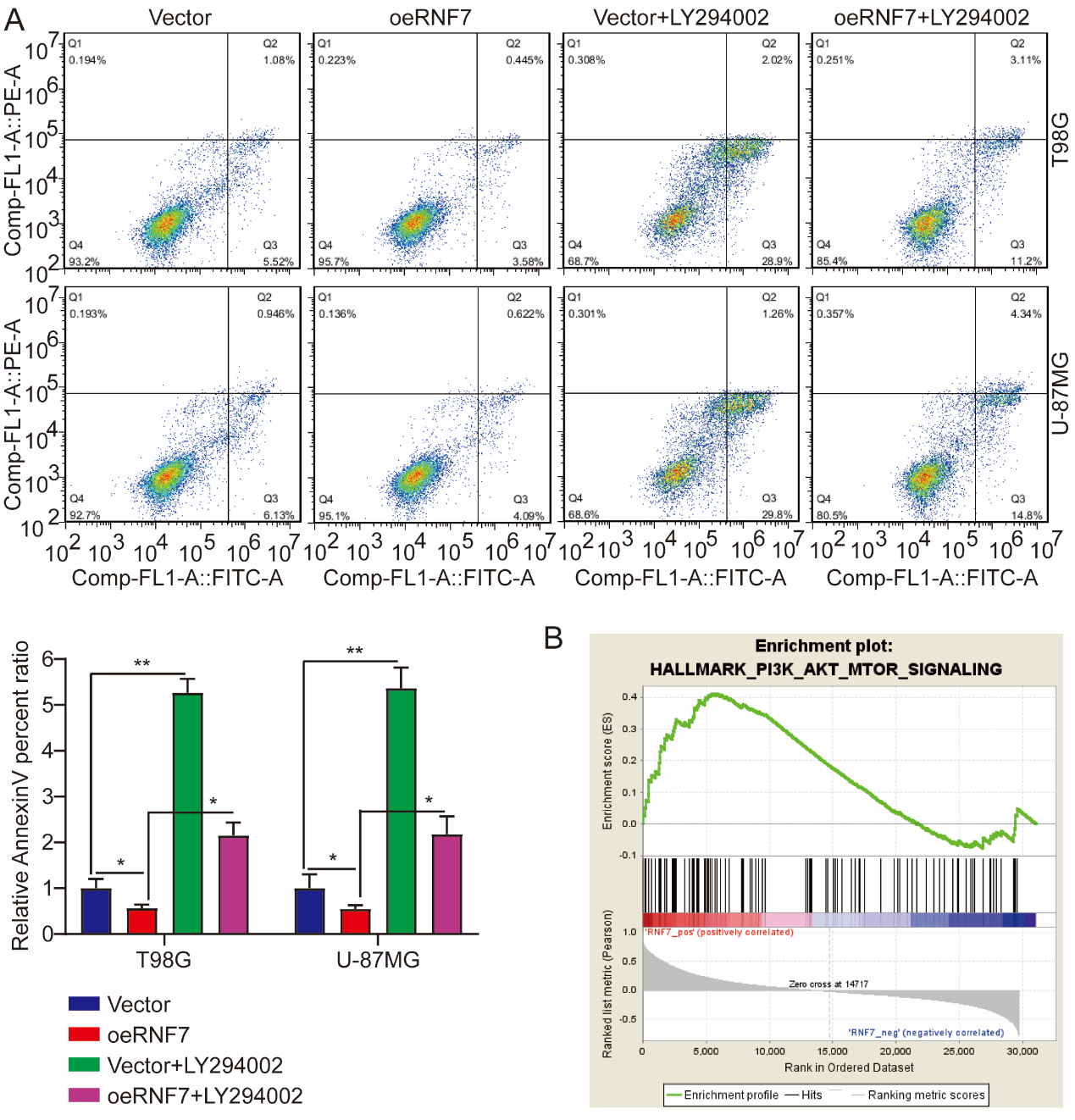


**Fig. S5 A.** The percentages of cell death, late apoptosis, early apoptosis, and live cells were calculated in different treatment groups. **B.** GSEA was performed using the GSEA software.


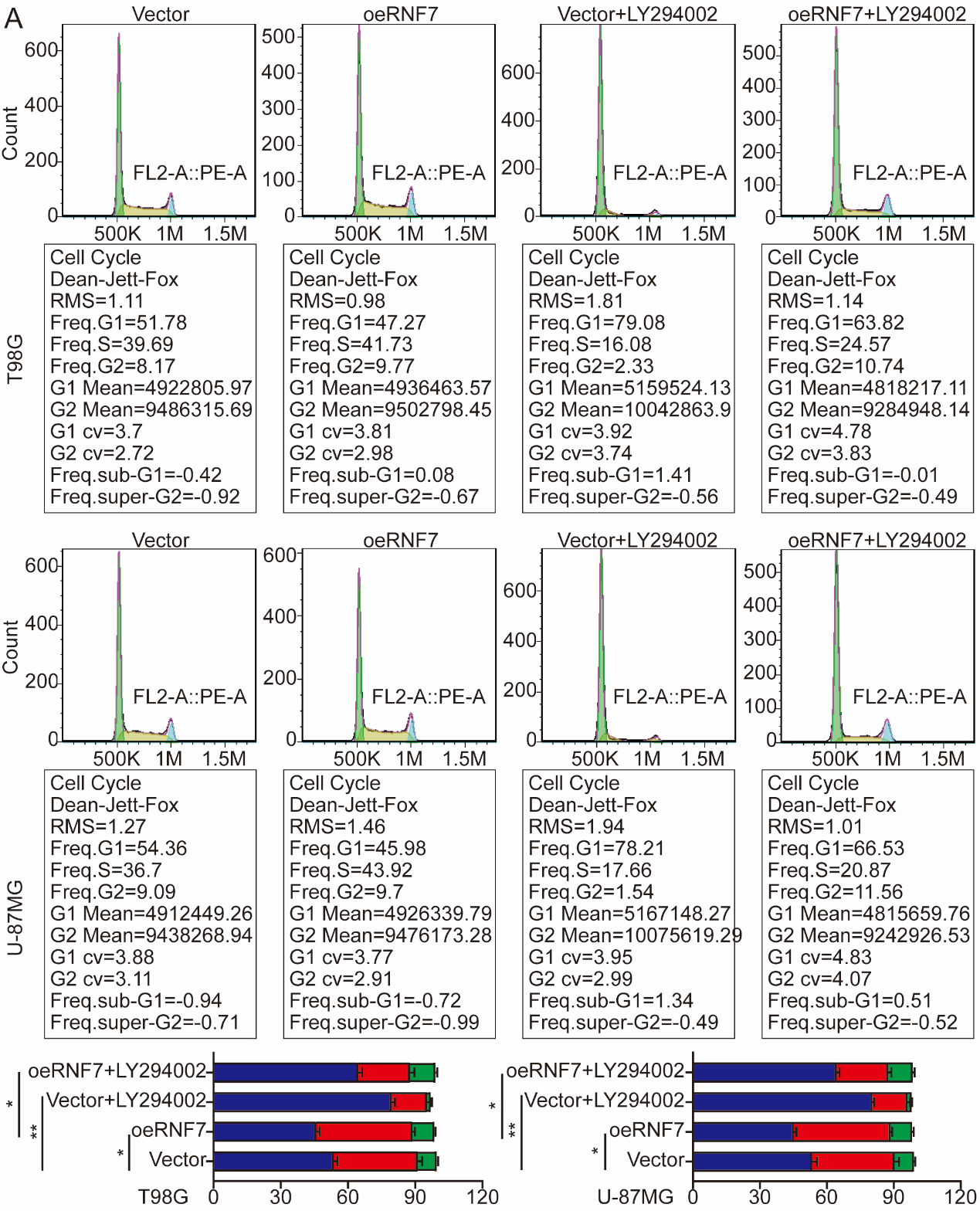


**Fig. S6 A.** RNF7 overexpression could promote cell cycle progression and the effect could be attenuated by treatment with PI3K/AKT signaling pathway inhibitors LY294002. Data were presented as mean ± s.d from three independent experiments. * *P* < 0.05 and ** *P* < 0.01.


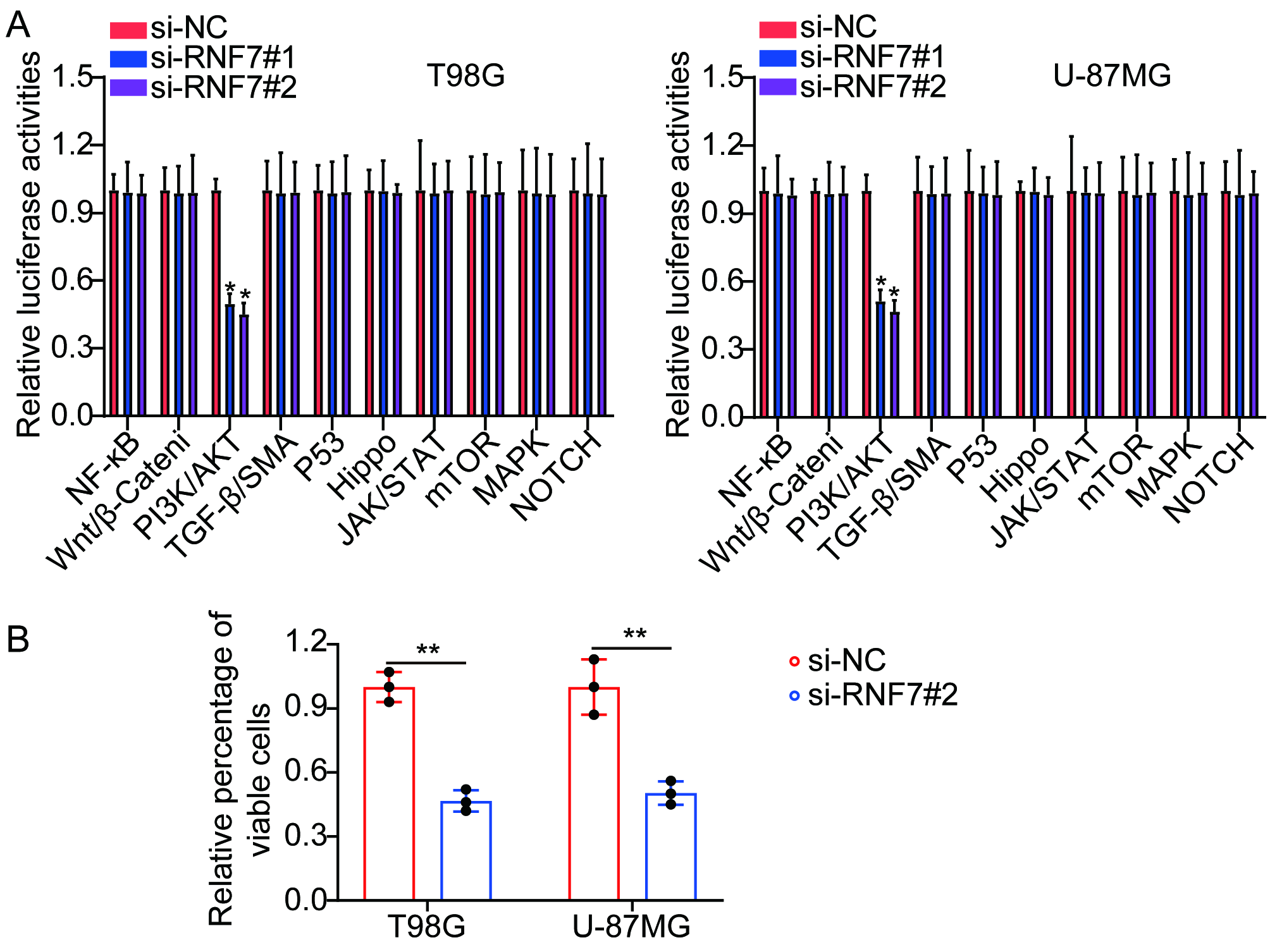


**Fig. S7 A.** RNF7 knockdown decreased the luciferase activities of PI3K/AKT by the dual luciferase reporter assay. Others remain unchanged.

**B.** Typical histogram. *p < 0.05 and **p < 0.01.
